# Gateway Entry Vector Library of Wolbachia pipientis Candidate Effectors from Strain *w*Mel

**DOI:** 10.1128/MRA.00806-18

**Published:** 2018-07-12

**Authors:** Irene L. G. Newton, Kathy B. Sheehan

**Affiliations:** a Department of Biology, Indiana University, Bloomington, Indiana, USA; Queens College

## Abstract

Wolbachia pipientis is an intracellular symbiont that modifies host biology using a type IV secretion system to inject bacterial effectors into the host cytoplasm. We utilized a bioinformatics approach to predict *Wolbachia* effectors and cloned the candidates into an entry vector, which can be utilized for subsequent analyses.

## ANNOUNCEMENT

Wolbachia pipientis is the most prevalent infection on Earth and is increasingly promoted for its use in disease vector control (1). Due to both the direct effects that Wolbachia may have on the transmission of human pathogens (2) and the myriad effects Wolbachia has on insect populations (3), it is important that we identify the mechanisms for symbiosis between Wolbachia spp. and their hosts. Although the type IV secretion system has long been hypothesized to be involved in host interaction (4), we conducted the first large-scale screen for effector proteins likely used by Wolbachia to manipulate host cell biology (5). Our research generated a set of candidate effectors, publicly available as a resource for further studies. The generation of the plasmid library is described as follows.

Wolbachia open reading frames from the *w*Mel genome were subjected to a BLAST search against the NCBI nr database (accessed April 2012) using TBLASTN v2.2.25+ with default options. In addition, we also performed a search of the Pfam-A database (v26.0) using hmmscan v3.0 with default options (http://hmmer.org), identifying Wolbachia proteins with homologies to domains enriched for eukaryote membership. In addition to proteins with eukaryotic homologies, we also included Wolbachia proteins specific to the genus. We then culled the proteins that were predicted to be made up of <200 amino acids in order to enrich the data set for true open reading frames.

**FIG 1:**
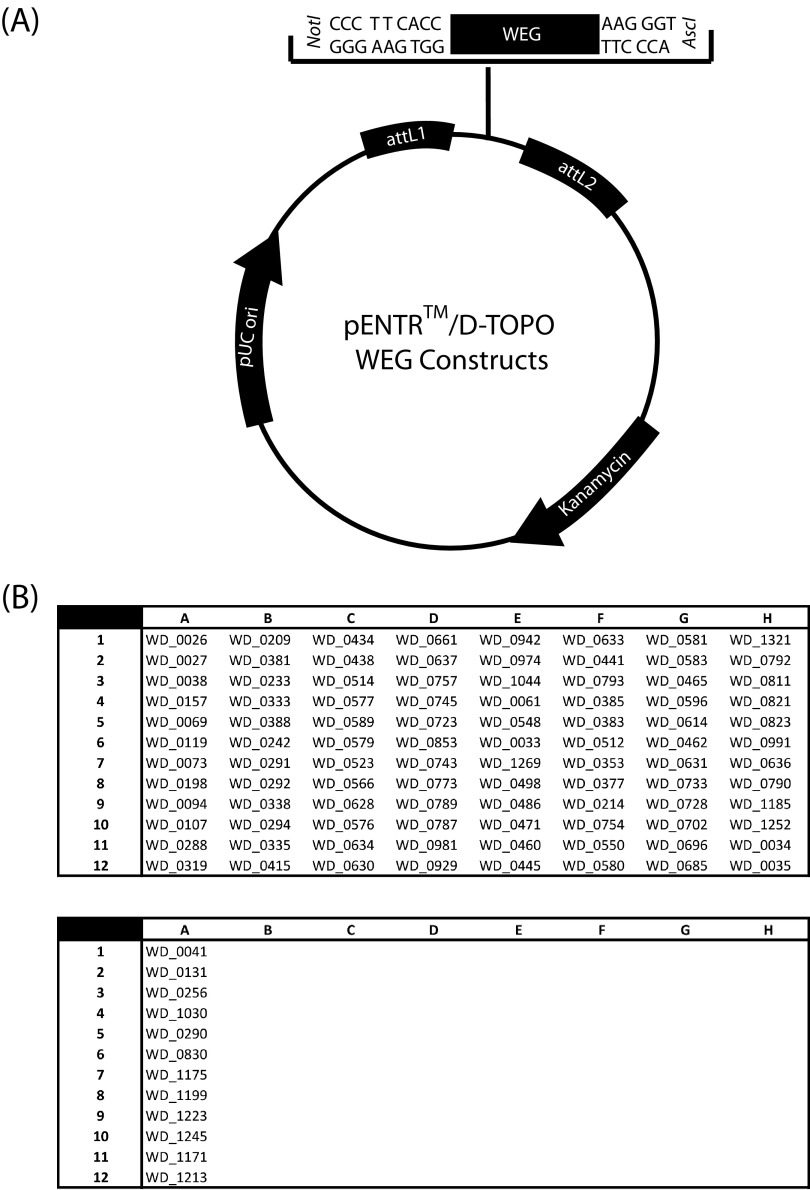
(A) Plasmid map for pENTRTM/D-TOPO (Invitrogen) constructs containing *Wolbachia* eukaryote-like genes (WEGs) and (B) organization of insert library with *Wolbachia* WEG accession numbers indicated.

We targeted the resulting 164 loci from the *w*Mel genome for amplification using modified forward primers to facilitate cloning by means of the Invitrogen Gateway pENTR/D-TOPO system (see reference 5 for more detail). As described in the user manual, blunt-end PCR products were directionally cloned into the pENTR/D-TOPO vector using the TOPO cloning reaction (Fig. 1A) and transformed into Invitrogen One Shot Top10 chemically competent E. coli cells using standard protocols. Transformants were plated on selective plates containing LB medium supplemented with kanamycin **(**LB_kan_). Colonies were selected and positive transformants were sequence verified to confirm that the protein products were in frame and correctly cloned.

A total of 108 pENTR/D-TOPO clones (in 100 µl of LB_kan_ with 25% glycerol) are included in the plasmid library on two 96-well plates (see Fig. 1B for insert accession numbers and locations on plates). Plates are stored at −80°C.

### Data availability.

Requests for the resource should be directed to the corresponding author, Irene L. G. Newton (irnewton@indiana.edu).
